# Floral Associations of Cyclocephaline Scarab Beetles

**DOI:** 10.1673/031.013.10001

**Published:** 2013-10-01

**Authors:** Matthew Robert Moore, Mary Liz Jameson

**Affiliations:** Wichita State University, Department of Biological Sciences, 1845 Fairmount, Wichita, KS, USA 67260-0026

**Keywords:** Cantharophily, Scarabaeidae, Dynastinae, Araceae, Arecaceae, Annonaceae, Nymphaceae

## Abstract

The scarab beetle tribe Cyclocephalini (Coleoptera: Scarabaeidae: Dynastinae) is the second largest tribe of rhinoceros beetles, with nearly 500 described species. This diverse group is most closely associated with early diverging angiosperm groups (the family Nymphaeaceae, magnoliid clade, and monocots), where they feed, mate, and receive the benefit of thermal rewards from the host plant. Cyclocephaline floral association data have never been synthesized, and a comprehensive review of this ecological interaction was necessary to promote research by updating nomenclature, identifying inconsistencies in the data, and reporting previously unpublished data. Based on the most specific data, at least 97 cyclocephaline beetle species have been reported from the flowers of 58 plant genera representing 17 families and 15 orders. Thirteen new cyclocephaline floral associations are reported herein. Six cyclocephaline and 25 plant synonyms were reported in the literature and on beetle voucher specimen labels, and these were updated to reflect current nomenclature. The valid names of three unavailable plant host names were identified. We review the cyclocephaline floral associations with respect to inferred relationships of angiosperm orders. Ten genera of cyclocephaline beetles have been recorded from flowers of early diverging angiosperm groups. In contrast, only one genus, *Cyclocephala*, has been recorded from dicot flowers. Cyclocephaline visitation of dicot flowers is limited to the New World, and it is unknown whether this is evolutionary meaningful or the result of sampling bias and incomplete data. The most important areas for future research include: 1) elucidating the factors that attract cyclocephalines to flowers including floral scent chemistry and thermogenesis, 2) determining whether cyclocephaline dicot visitation is truly limited to the New World, and 3) inferring evolutionary relationships within the Cyclocephalini to rigorously test vicarance hypotheses, host plant shifts, and mutualisms with angiosperms.

## Introduction

**Figure 1.**
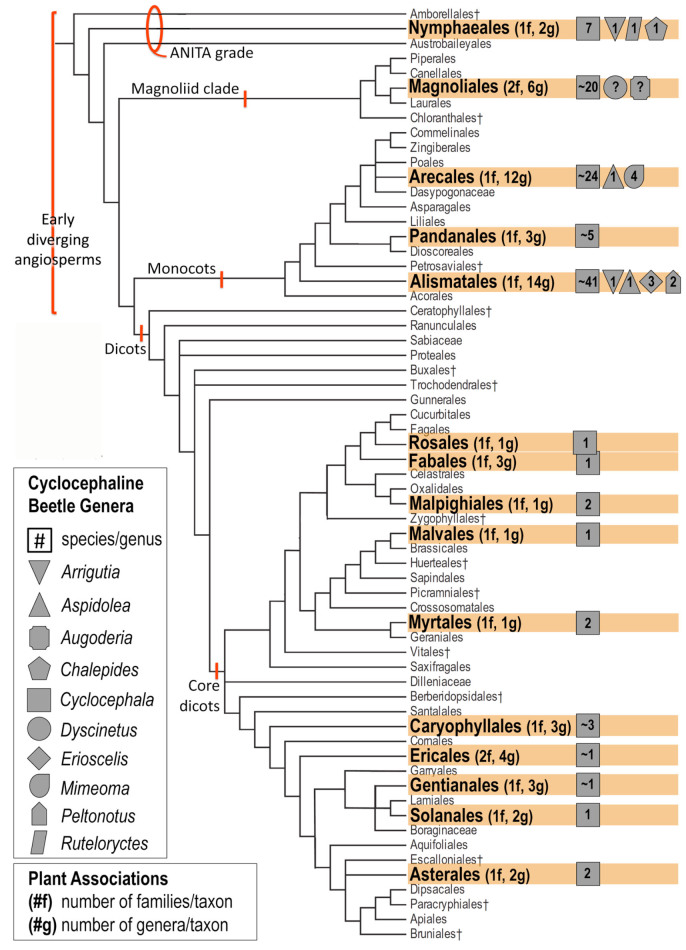
Cyclocephaline beetle genera and their associations with angiosperm plant lineages (plant phylogeny from APGIII 2009). Icons denote beetle genera that are associated with angiosperm plant lineages. Numbers in the icons indicate the number of species for each beetle genus. If the number of beetle species is unresolved due to conflict in the literature, this is indicated with ~ symbol (the number may be × ± I species). If the beetle genus has not been satisfactorily associated with the plant lineage, it is denoted with a ? symbol. For each angiosperm plant lineage, the number of families and genera that the beetles are associated with is denoted with #f (number of families) and #g (number of genera). See Appendix I for data. High quality figures are available online.

The Cyclocephalini (Coleoptera: Scarabaeidae: Dynastinae) is the second largest rhinoceros beetle tribe, currently containing 15 genera and nearly 500 described beetle species (Jameson et al. 2002; Ratcliffe 2003; Smith 2006). Cyclocephalines have a pantropical distribution, though the majority of the group's generic and species diversity is concentrated in the New World (Ratcliffe 2003; Ratcliffe and Cave 2006). Most genera are sexually dimorphic, with males having enlarged protarsal claws and females having expanded elytral epipleura (Moore 2012). Cyclocephalines are important economically and ecologically as root pests (larvae) and pollinators (adults) (Ratcliffe 2003; Ratcliffe and Paulsen 2008). Adult cyclocephaline beetles can be found within the inflorescences of early diverging angiosperm groups (the family Nymphaeaceae, magnoliid clade, and monocots; Figure 1) and have been shown to contribute to pollination in the Annonaceae, Araceae, Arecaceae, Cyclanthaceae, Magnoliaceae, and Nymphaeaceae (Cramer et al. 1975; Beach 1982; Beach 1984; Young 1986; Young 1988b; Gottsberger 1989; Dieringer et al. 1999; Hirthe and Porembski 2003; Maia et al. 2012). Studies of these interactions indicate that some early diverging angiosperm groups offer rewards to cyclocephalines in the form of mating sites, food, and metabolic boosts associated with floral thermogenicity in return for pollination services (Gottsberger 1986; Young 1986; Seymour et al. 2009). Cyclocephaline visitation of dicot flowers is poorly known and little studied.

Cyclocephaline floral associations have been reported in journals, books, and monographs since the late 18^th^ century. However, the prevalence, geographic scope, and biological importance of these records are difficult to gauge because publications summarizing cyclocephaline floral visitation are somewhat dated and report floral visitation only for specific plant families, geographic areas, or vegetation types (Henderson 1986; Gibernau 2003; Gottsberger and Silberbauer-Gottsberger 2006; Gibernau 2011). The fragmentary nature of these data and the citation of unpublished observations have hampered the ability to identify floral association trends within cyclocephaline genera and species.

The phylogeny of the Cyclocephalini was investigated for the first time by Clark (2011), and the generic-level relationships within the tribe remain an area of active research by M. R. Moore. Tribal circumscription of the Cyclocephalini is subject to change based on ongoing phylogenetic analyses. This research will provide an evolutionary framework for interpreting patterns of floral visitation. Compilation and synthesis of a checklist of floral associations is needed in order to understand the ecology of the Cyclocephalini within a phylogenetic context.

This checklist synthesizes data (plant and beetle species, geographic locality, and original citation) for the floral associations of adult cyclocephaline beetles. Invalid nomenclature in the surveyed literature is identified and corrected; conflicting data, sources of error, and uncertainty in the data are identified; and unpublished floral association data from examined voucher specimens are added. The aim of this work is to promote future research of these ecological interactions by providing a comprehensive data set of the taxonomic and geographic scope of floral visitation for cyclocephaline beetles.

## Materials and Methods

Literature was surveyed from 1758 (Linnaeus) to 2012. Keyword searches for all cyclocephaline genera (*sensu* Ratcliffe and Cave 2006; Clark 2011) were conducted in the following databases: BioOne® (www.bioone.org), BIOSIS Previews® (http://apps.webofknowledge.com/), JSTOR (www.istor.org), and Biodiversity Heritage Library (www.biodiversitvlibrary.org). Every host plant reference from Pike et al. (1976) was checked for floral association data.

All reported cyclocephaline species names from the literature were verified by referencing the original species description and monographic treatments of the Dynastinae (Endrödi 1985; Ratcliffe 2003; Ratcliffe and Cave 2006). Synonyms or misspelled cyclocephaline species names in the literature were updated to reflect current nomenclature. All reported host plant names were verified using the peer-reviewed botanical taxonomic databases Tropicos (www.tropicos.org) and The Plant List (www.plantlist.org). Synonyms or misspelled plant names were updated to reflect current nomenclature based on The Plant List (2010). In some cases, scientific names in the literature could not be identified as valid or invalid (e.g., unavailable manuscript names or conflicting synonyms). Some unverified plant names were reported according to the original citation for the floral association, and the name was noted as unresolved. Occasionally, host plant and beetle species were not assigned an author in the reference for an association. This caused problems due to the prevalence of synonyms and homonyms in the plant and insect literature. Resulting ambiguities were rectified to the extent possible and explained in the remarks column (Appendix 1).

Borrowed specimens of cyclocephaline species allowed for direct evaluation of specieslevel identifications that were reported by several authors. Particularly, this included specimens of *Cyclocephala sexpunctata* Laporte (1840) and *C. brevis* Höhne (1847) collected by George Schatz, Helen Young (La Selva Biological Station, Costa Rica), Alberto Seres, and Nelson Ramirez (Henri Pittier National Park, Venezuela), with floral association data that were subsequently published or unpublished. Identifications of these specimens (or specimen vouchers) were critically examined (Moore 2011). Exemplar material borrowed from the University of Nebraska State Museum (authoritatively identified by B. C. Ratcliffe) and monographic treatments (Ratcliffe 2003; Ratcliffe and Cave 2006) served as the basis for evaluating species identifications as well as detailed images of some type specimens. The operating assumption was that the collectors and authors were consistent with their species-level determinations. Identifications deemed incorrect based on current taxonomy were updated and noted accordingly. Unpublished host plant data were also found with cyclocephaline specimens in collections. These specimens were collected by M. R. Moore and deposited at Wichita State University, Wichita, Kansas, USA, or loaned from the following institutions:

INBC: Instituto Nacional de Biodiversidad, Santo Domingo de Herédia, Costa Rica (Angel Solis)
MLUH: Zentralmagazin Naturwissenschaftlicher Sammlungen, Martin Luther Universität Halle-Wittenberg, Halle, Saxony-Anhalt, Germany (Karla Schneider)
MNHN: Muséum national d'Histoire naturelle, Paris, France (Olivier Montreuil)
SEMC: Snow Entomological Museum, University of Kansas, Lawrence, KS (Zach Falin and Jennifer Thomas)
UNSM: University of Nebraska State Museum, Lincoln, NE (Brett Ratcliffe and Matt Paulsen)
USNM: U.S. National Museum, Washington, D.C. (currently housed at the University of Nebraska State Museum for off-site enhancement) (Floyd Shockley and Dave Furth)
UVGC: Universidad del Valle de Guatemala, Guatemala City, Guatemala (Jack Schuster and Enio Cano)
WICH: Wichita State University, Wichita, KS (Mary Liz Jameson)
ZMHB: Museum für Naturkunde der Humboldt Universität zu Berlin, Berlin, Germany (Johannes Frisch and Joachim Willers)

Concrete and anecdotal evidence of floral associations were also included in the checklist. The nature of the published association occasionally needed clarification or elaboration (e.g., cyclocephalines reported near flowers but not on them or museum specimens covered in resin and pollen). These clarifications were provided in the remarks column of Appendix 1. A large amount of unpublished and inaccessible data exists with regard to cyclocephaline floral visitation. These records provide ambiguous data for plant species, cyclocephaline species, locality, and associated voucher information. For example, Schatz (1990, Table 7.3) recorded known and predicted (without distinguishing the two) plant taxa pollinated by dynastines in the Neotropics. Schatz (1990, Table 7.4) recorded cyclocephaline plant visitation at La Selva Biological Station, but a large amount of data could not be extracted because of the nonspecific nature of the record (i.e., the data were reported at the tribal-level rather than at the species-level). These inaccessible data are important because they report certain associations that are not recorded elsewhere in the literature. Repetitive data from these types of records were omitted from the checklist. Only unique generic or species-level plant associations were reported for the beetle tribe from these data sets. These non-specific records are reported at the end the checklist with the intention that they be reevaluated with the addition of more data.

**Table 1.**
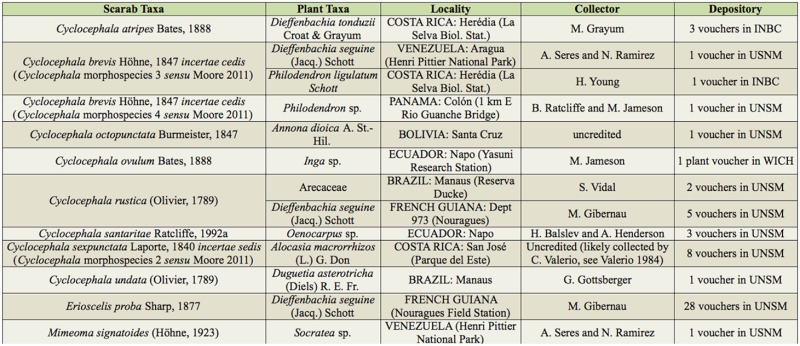
Previously unpublished cyclocephaline beetle floral association data.

## Results

Based on species-specific records from the literature and voucher label data, at least 97 cyclocephaline species from nine or 10 genera (depending on the identity of the cyclocephaline reported by Gibbs et al. (1977)) were recorded in association with the flowers of at least 161 species representing 58 genera, 17 families, and 15 orders (Appendix 1). Examined voucher specimens occasionally had unique, unpublished, floral association data. Thirteen new plant associations are provided in Table 1. Examined voucher specimens that did not have unique data are noted in Appendix 1. The most specific data are summarized at the generic-level for the plant association (plant classification according to the Angiosperm Phylogeny Group III (2009)) in Table 2 and are provided in full detail (lowest-level taxonomy, geographic data, and references) in Appendix 1. Cyclocephaline beetle genera and their associations with angiosperm plant lineages were mapped onto the APG III angiosperm phylogeny (Figure 1).

Five of the 15 cyclocephaline genera were not reported as floral visitors in any of the surveyed literature: *Acrobolbia* Ohaus (1912), *Ancognatha* Erichson (1847), *Harposcelis* Burmeister (1847), *Stenocrates* Burmeister (1847), and *Surutu* Martínez (1955). Preliminary phylogenetic analysis of the Cyclocephalini indicated that the Neotropical genus *Parapucaya* Prell (1934) (Dynastinae: Pentodontini) and the Indonesian archipelago genus *Neohyphus* Heller (1896) (Dynastinae: Oryctoderini) fall within a potential newly defined Cyclocephalini (Clark 2011). These genera were included in the systematic literature searches but yielded no floral association records. The results of Clark (2011) hypothesized that the genus *Erioscelis* Burmeister (1847) is sister to all remaining genera of the Cyclocephalini + *Neohyphyus + Parapucaya. Erioscelis* was included in this checklist because of its documented visitation of several genera in the Araceae (also visited by other cyclocephalines) and its historical inclusion in the Cyclocephalini.

**Table 2.**
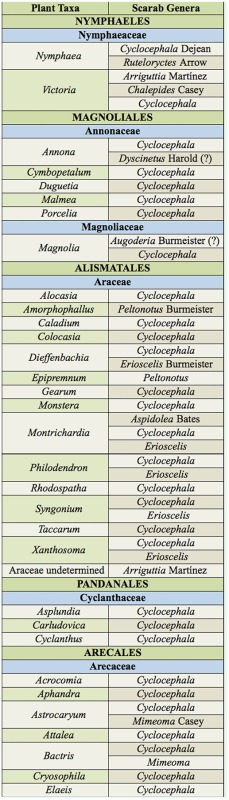
Generic-level summary of floral association records for the Cyclocephalini (group names in parentheses are based on APG III (2009)) [? indicates a potentially dubius record, see Appendix I].

**Continued.**
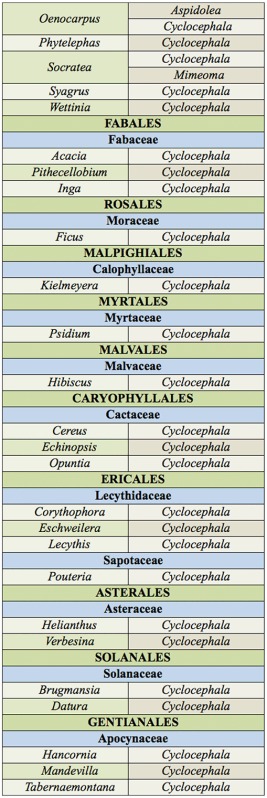

Floral associations that are less specific or ambiguous (non-specific records) were also reported (Appendix 1). For example, Listabarth (1996) reported dynastine scarabs, with no further species identification, on three species of *Bactris* palms (Arecales). These data include records for Scarabaeidae, Dynastinae, and beetles on flowers that fit the general pattern of cyclocephaline floral visitation (nocturnal visitation of bowl-shaped, thermogenic inflorescences). Non-specific records were included in the checklist with the hope that they may be reevaluated with additional data.

Gathering and interpreting floral association data were complicated by the prevalancy of synonyms, invalid names, and unavailable names in the literature. Based on The International Code of Zoological Nomenclature (ICZN 1999), an unavailable name is a name that is excluded from use due to the requirements of the code. For example, the unavailable name *Cyclocephala inpunctata* was reported in the surveyed literature (Gottsberger 1986, 1988). *C. inpunctata* has never been described in the literature. This name is unavailable and was likely reported in error. Based on published locality data for the floral association, images of the beetle (Gottsberger 1988; Figure 4a, 5 a–d), and subsequently published records, we consider this species to be *Cyclocephala quatuordecimpunctata* Mannerheim (1829) (personal communication with B. C. Ratcliffe, April 2011). Synonyms of six cyclocephaline genus or species names were reported in the surveyed literature; these invalid names were updated based on current nomenclature (Appendix 2). Synonyms of 25 plant genus or species names were reported in the surveyed literature and on voucher specimen label data; these invalid names were updated based on current nomenclature (Appendix 3).

Seven unresolved or unavailable plant names were reported from label data and in the surveyed literature (Appendix 4). According to The Plant List (2010), unresolved names are those for which “it is not yet possible to assign a status of either ‘accepted’ or ‘synonym.’” Two of these names, *Philodendron atlanticum* and *Dieffenbachia longivaginata*, were unavailable manuscript names (place-holder names for species that were later described) of Thomas Croat and Michael Grayum (Missouri Botanical Garden, St. Louis, Missouri, USA). These species were identified as *Philodendron ligulatum* Schott and *Dieffenbachia tonduzii* Croat and Grayum, respectively (personal communication with T. Croat and M. Grayum, April 2011). *Xanthosoma macrorrhizas* i s an unavailable name that was reported by Valerio (1984). This species may be the cultivated, naturalized, non-native species *Alocasia macrorrhizos* (L.) G. Don (personal communication with T. Croat, April 2011).

Certain cyclocephaline species were commonly reported as floral visitors. For example, *Cyclocephala sexpunctata* had over 20 floral visitation records in the surveyed literature (Appendix 1). *C. sexpunctata* is externally nearly identical to *C. brevis* (*sensu* Ratcliffe 2003; Ratcliffe and Cave 2006). Research on these two species showed that they represent four, or potentially five, morphospecies (Moore 2011). This conclusion was based on male genitalic characters, the form of the female epipleuron, and extensive range and spatial data (Moore 2011). The taxonomy of the species *C. sexpunctata* and *C. brevis* remains unresolved (a possible species complex), and their floral associations were reported in detail (Moore 2011). Some voucher specimens for reported floral associations of *C. sexpunctata* and *C. brevis* remain to be examined, and some data will require reinterpretation after the examination of type specimens.

## Discussion

Examination of cyclocephaline floral associations with respect to inferred relationships of angiosperm orders revealed that 10 of the 15 genera of cyclocephaline beetles have been recorded from flowers of early diverging angiosperm groups (the family Nymphaeaceae, magnoliid clade, and monocots; Figure 1). In contrast, only one genus, *Cyclocephala*, has been recorded from dicot flowers (Figure 1). Experimental and observational studies have demonstrated that cyclocephalines can act as pollinators in Nymphaeales, Magnoliales, Arecales, Pandanales, and Alismatales (Figure 1; Table 2) (Cramer et al. 1975; Beach 1982; Beach 1984; Young 1986; Young 1988b; Gottsberger 1989; Dieringer et al. 1999; Hirthe and Porembski 2003; Maia et al. 2012). In these early diverging plant groups, a wide set of floral traits and floral pollination syndromes indicate a correlation with cyclocephaline beetles (large pollen grains with sticky exudates, sturdy and funnel-shaped inflorescences or large disc-shaped flowers, timing of anthesis, and therm ogenesis) (Thien et al. 2009; Gibernau et al. 2010). These angiosperm orders offer rewards to cyclocephalines in the form of mating sites, food, and heat resources associated with floral thermogenicity (Young 1986; Seymour et al. 2009).

Some cyclocephaline/flower associations are mutualistic (Cramer et al. 1975; Beach 1982; Beach 1984; Young 1986; Young 1988b; Gottsberger 1989; Dieringer et al. 1999; Hirthe and Porembski 2003; Maia et al. 2012). Ervik and Knudsen (2003) provide a compelling argument that scarab pollination of the Nymphaeaceae (Nymphales) is a mutualistic relationship that dates to the early Cretaceous. Whether this represents an example of coevolution is unclear, and only one study has addressed this hypothesis (Schiestl and Dötterl 2012). Schiestl and Dötterl (2012) argued that volatile organic compound production/detection systems arose in the Scarabaeoidea during the Jurassic, whereas floral volatile organic compounds arose in the Cretaceous/Paleocene. This was taken as evidence that early diverging angiosperm plant/scarab associations evolved due to a preexisting sensory bias in scarabs rather than as a result of coevolution (Schiestl and Dötterl 2012). However, coevolution could not be ruled out for the mutualism between cyclocephaline scarabs and aroid flowers (Schiestl and Dötterl 2012).

Floral visitation of the core eudicot clade (Figure 1) by cyclocephalines is poorly described and, in certain cases, differs significantly from a pollination mutualism. Such cases involve feeding and mating within flowers in which cyclocephalines have no apparent pollinating function and may destroy the reproductive capability of the plant. For example, in the Brazilian dicot *Opuntia monocantha* Haw. (Caryophyllales), *Cyclocephala* have been observed mating within the flowers and feeding on stamens (Lenzi and Inácio Orth 2011). Observations made on *Echinopsis ancistrophora* Speg. subsp. *ancistrophora* (Caryophyllales) flowers indicate that *Cyclocephala* visitors display destructive feeding behavior and do not contribute to reproduction (Schlumpberger et al. 2009). *Cyclocephala metrica* Steinheil (1874) was observed feeding on seeds in flower heads of *Verbesina encelioides* (Cav.) Benth. and Hook. f. ex A. Gray (Asterales) in Argentina (Hayward 1946). Seed predation in phytophagous scarabs is rare, the only other known example being some members of the subtribe Anisopliina (Scarabaeidae: Rutelinae: Anomalini) that feed on grass seeds (Poaceae) (Jameson et al. 2007).

In contrast to apparent destructive associations with dicots, only one detailed account provides evidence of a cyclocephaline beetle pollinating a eudicot. Prance (1976) observed male and female *Cyclocephala verticalis* Burmeister (1847) occupying the inflorescences of *Le cy this, Corythophora,* and *Eschweilera* (Ericales) in Amazonas, Brazil. *C. verticalis* was strong enough to lift the closed androphore flap of Lecythidaceae (Encales) inflorescences and displayed selective feeding of floral parts, eating only staminode tissue at the apex of the androphore and leaving fertile stamens untouched (Prance 1976). Based on these observations, *C. verticalis* was considered a likely pollinator of some Lecythidaceae genera, though this hypothesis was not tested (Prance 1976).

Gottsberger (1986) considered cyclocephaline floral visitation of the dicot families Apocynaceae (Gentianales), Calophyllaceae (Malpighiales), and Sapotaceae (Ericales) to be opportunistic. In the absence of early diverging angiosperm host flowers, Gottsberger (1986) hypothesized that cyclocephalines would visit strongly scented flowers of other groups. Cyclocephalines have been shown to aggregate based on floral scent compounds alone (Gottsberger et al. 2012). Cyclocephaline species (and populations) likely are biased towards a wide range of floral scent compounds. Eudicot species with geographically variable floral scent profiles may evolve scents that incidentally stimulate cyclocephaline aggregation by randomly sampling the sensory bias range of scarabs present in that area (e.g., Schlumpberger and Raguso 2008; Schlumpberger et al. 2009). This scenario, if accurate, would lend support to the hypothesis of Schiestl and Dötterl (2012) that preexisting sensory biases in cyclocephalines have an important role in determining the host flower profile of a given cyclocephaline species.

Based on the assembled data (Appendix 1), cyclocephaline visitation of eudicots is limited to the New World. It is unknown whether this shift represents an evolutionary event that occurred in New World cyclocephalines. Observations of cyclocephalines on dicot flowers (Figure 1) have largely been made by chance and have not been the subject of rigorous experimentation or sampling protocols. Thus, it is quite possible that Old World cyclocephalines (*Ruteloryctes, Peltonotus*, and potentially *Neohyphus*) visit both early diverging angiosperm groups and dicot groups, but dicot associations have not been recorded. However, it is certain that the known diversity of host flowers lineages is much higher for New World cyclocephalines (15 orders, 17 families, and 58 genera) compared to Old World cyclocephalines (two orders, two families, and three genera) (Appendix 1). This correlation may indicate that the radiation of the cyclocephalines in the New World was accompanied by a subsequent increase in the diversity of their floral associations.

Cyclocephaline species are generally oligophagous or polyphagous. For cyclocephaline species with multiple host records, only seven species have been recorded from a single host plant genus (monophagous), 23 species have been reported from multiple host plant genera within a family (oligophagous), and 27 species have been recorded from multiple host plant families (polyphagous) (Appendix 1). Single inflorescences often contain multiple cyclocephaline species, and an extreme example is *Dieffenbachia nitidipetiolata* Croat and Grayum (Alismatales), which was visited by at least nine *Cyclocephala* species at La Selva Biological Station, Costa Rica (Young 1990; see Croat 2004 for plant identification). These multi-species aggregations might be explained if floral scents are serving as sex pheromones for multiple cyclocephaline species (Schatz 1990). This hypothesis may be supported by the observations of Gottsberger et al. (2012) that *Cyclocephala literata* Burmeister will aggregate due to floral scent compounds alone.

The consequences of polyphagous and oligophagous cyclocephalines for pollination efficiency have been experimentally addressed, indicating that cyclocephaline floral visitors are differentially important as pollinators due to an interaction between their relative abundance and specific behavior (Young 1986, 1988a, b, 1990). It is less clear how cyclocephalines species, which often mate inside inflorescences, maintain sexual isolation in close proximity to multiple congenerics. A single infloresence may host large crowds of beetles, often more then 30 individuals (Maia et al. 2012). Sexual isolation may be maintained due to interspecific mating morphology (Moore 2012). Sexually dimorphic cyclocephaline species have enlarged protarsal claws (males), and the elytral epipleuron variably expanded into a shelf or flange (females). Morphological differences among epipleural expansions are useful for species-level identification in the Cyclocephalini (Ratcliffe 2003). Females have sclerotized patches, sometimes with setae, on the ventral portion of epipleural expansions (Moore 2012). It is hypothesized that the interaction between the male protarsal claw, the female epipleural expansions, and the ventral portion of the female elytra serves as a pre-copulatory sexual isolation mechanism. Further sexual isolation between species is accomplished by species-specific differences in male genitalic structure (Moore 2012). The male protarsal claw and the female epipleuron may also be involved in intraspecific mate competition. For example, male *Cyclocephala gravis* Bates were observed clinging tightly to the epipleural structures of a female (guarding behavior), thus limiting the mating access of other C. *gravis* males (Moore 2012). Cyclocephaline beetles exhibit some similarity to hopliine scarabs (Scarabaeidae: Rutelinae: Hopliini), which are generalist flower visitors in South Africa (Ahrens et al. 2011). Sexual dimorphism has evolved independently several times within the Hopliini (Ahrens et al. 2011). Evolution of sexual dimorphism in hopliines could be tied to the group's biology, as they feed and compete for mates within inflorescences (Midgeley 1992; Ahrens et al. 2011). Sexual dimorphism in cyclocephalines and hopliines may be analogous, driven by selection pressures related to oligophagous and polyphagous flower feeding, mating behavior, and host visitation.

Cyclocephaline beetles and floral associations provide an ideal system for investigating ecology (pollination, competition) and evolution (sexual selection, mutualisms). A wellfounded phylogenetic framework for the Cyclocephalini is needed to advance this work. While ecological associations between beetles and early diverging angiosperm groups is fairly well-established, additional research is necessary to understand the ecological and historical associations of cyclocephaline beetles and dicots. Specifically, research is needed to address the apparent cyclocephaline diversification on New World dicots. Research on cryptic species of host plants and beetles is fundamental to understanding this system. This includes the role of floral volatile compounds in attracting cyclocephaline beetles and patterns of pollination, herbivory, and interspecific competition within floral hosts.

## Figures and Tables

**Appendix 1.**
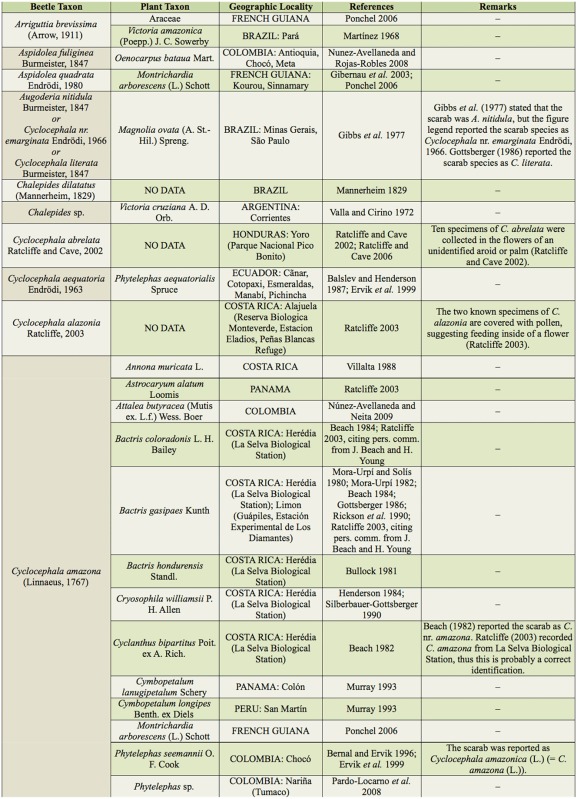
Checklist of floral associations for the Cyclocephalini (Scarabaeidae: Dynastinae).

**Appendix 2.**
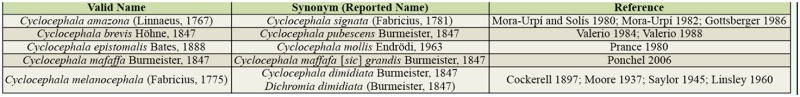
Cyclocephaline synonyms reported in the floral association literature.

**Appendix 3.**
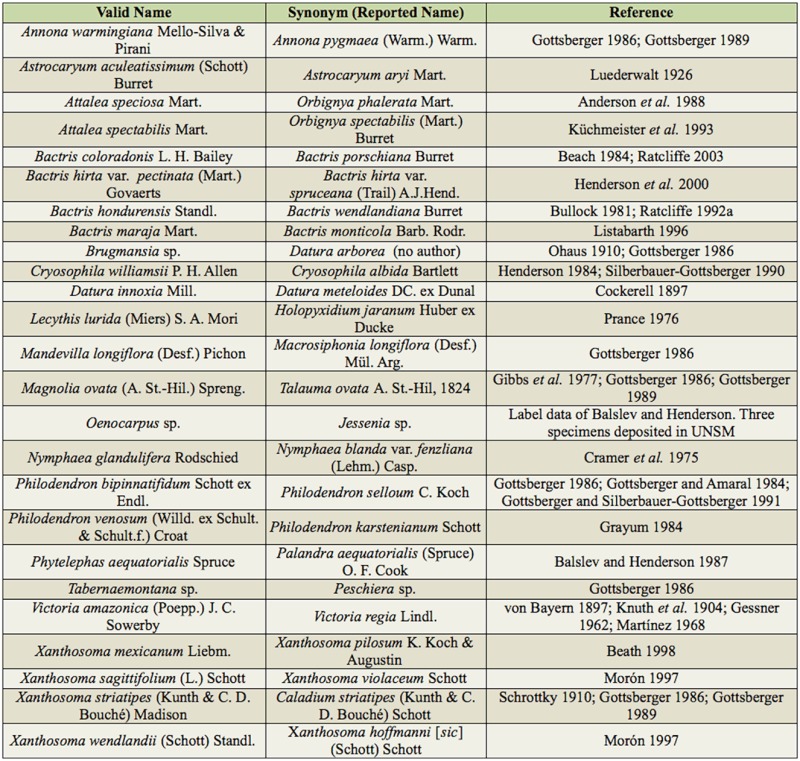
Plant synonyms reported in floral association literature and on voucher specimen label data.

**Appendix 4.**
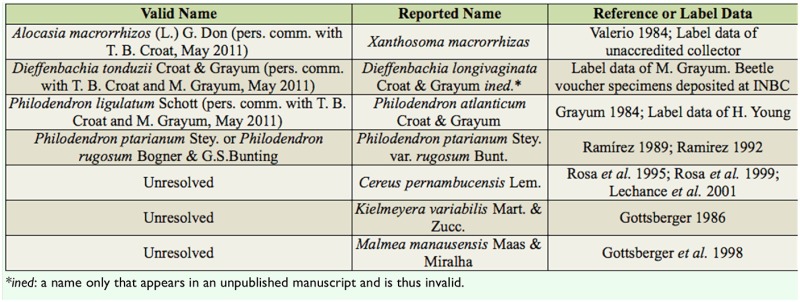
Unavailable and unresolved plant names from the floral association literature and voucher specimen label data.
